# 

**Published:** 1849-02

**Authors:** 


### WESTERN JOURNAL

OF

### MEDICINE AND SURGERY.

EDITOR.

---

LUNSFORD P. YANDELL, M. D.,

PROFESSOR OF CHEMISTRY AND PHARMACY IN THE UNIVERSITY OF LOUISVILLE.

To Subscribers.—We must again make an earnest appeal to the subscribers to the Western Journal of Medicine and Surgery to remit their dues. We are making heavy expenditures the present year, determined to keep it in the first rank of Medical periodicals. We hope that our subscribers will enable us to fulfil our intentions without embarrassment.

Feb. 1849. PRENTICE & WEISSINGER.

RECEIPTS OF MEDICAL JOURNAL FOR JANUARY, 1849.

| | |
|---|---|
| Drs. Woodson & Donnell, Franklin, Ind., | $5 00 |
| Dr. M Marsh, Indianapolis Ind., | 10 00 |
| C. W. Deane, Fayetteville, Ark., | 5 00 |
| J. S. Wallace, Springfield, Ind., | 15 00 |
| J. R. Buchanan, Cincinnati, O., | 5 00 |
| J. S. Lane, Clintonville, Ky., | 5 00 |
| O. F. Crothers, Utica, Ind.. | 5 00 |
| T. W Tavlor, Rossiclair, Ill., | 10 00 |
| ——— Kerrick, Salina, Ky., | 10 00 |
| E. C. Dicus, New Providence Tenn., | 10 00 |
| S. Rolin, Rankins, Tenn., | 5 00 |
| J. A. Nunn, Lanefield, Tenn., | 5 00 |
| T. Wathen, Uniontown, Ky., | 5 00 |
| J. P. Henderson, Newville, O., | 5 00 |
| C. J. Blackburn, Frankfort, Ky. | 5 00 |
| N. A. Page, Conyersville, Tenn. | 5 00 |
| J. S. Seaton, Jeffersontown, Ky., | 20 00 |
| W. H. Lee, Columbia. Mo., | 5 00 |
| L. Brown, Russellville, Tenn., | 5 00 |
| W A. Hickman, Bardstown, Ky., | 10 00 |
| J. E. Shipp, Centreville, Tenn., | 5 00 |
| ——— Brown, Brushy Fork, Ky., | 5 00 |
| ——— Keith, Merom, Ind. | 15 00 |
| J. A. Kinnebrew, Smithdale, Miss. | 5 00 |
| W. Kinney, Millersburg, Ky., | 5 00 |
| J. Edwards, Leavenworth, Ind. | 5 00 |
| A. B. Dodd, Biloxi, Miss., | 5 00 |
| T. J. Pollard, Fayetteville, Ark., | 10 00 |
| J. C. Cook, Newport, Ind. | 10 00 |
| W. C. Hendrick, Sullivan, Ill., | 2 00 |
| R. B. English, Howell Springs, Ky., | 5 00 |
| ——— Palmer, Harrisonville, Mo., | 2 50 |

